# The upper percolation threshold and porosity–permeability relationship in sandstone reservoirs using digital image analysis

**DOI:** 10.1038/s41598-022-15651-3

**Published:** 2022-07-04

**Authors:** Ryan L. Payton, Domenico Chiarella, Andrew Kingdon

**Affiliations:** 1 Clastic Sedimentology Investigation (CSI), Department of Earth Sciences, Royal Holloway, University of London, Egham, Surrey UK; 2 British Geological Survey, Keyworth, Nottingham, UK

**Keywords:** Stratigraphy, Geophysics, Sedimentology

## Abstract

Subsurface sandstone deposits represent globally ubiquitous reservoirs which can potentially provide the characteristics necessary for the effective geological storage of CO_2_. Geological carbon storage is widely agreed to be a key asset in tackling anthropogenic emissions and climate change to reach a sustainable ‘net zero’, despite the present financial challenges associated with it. Therefore, improved understanding of the characteristics of the materials in which we plan to store many gigatons of CO_2_ is critical. Developing cheaper characterisation techniques is therefore crucial to support the global push for net zero. In this work we use digital analysis of 3D microscale X-ray images of a range of sandstone samples to constrain the porosity–permeability relationship and the upper percolation threshold; the point at which near full pore structure connectivity is achieved. This is one of the most significant controls on the viability of carbon storage as a practical solution to achieving net zero. We find that the upper percolation threshold in sandstone occurs at ca. 14% total porosity whilst the relationship between porosity ($\phi$) and permeability ($K$) can be defined as $K={10}^{5.68} {\phi }^{3.88}$. The investigation of the upper percolation threshold may allow a target criterion to be designated when assessing potential carbon storage reservoirs, whilst investigation of the porosity–permeability relationship allows for a greater understanding of the fluid flow regimes in the subsurface. By using a digital technique to assess carbon storage reservoir potentiality we show that initial characterisation of reservoirs can be carried out rapidly and relatively economically, prior to further full reservoir characterisation studies. This approach is also non-destructive, allowing samples to be reused and multiple analytical phases performed on the same materials.

## Introduction

The increasing global urgency to tackle anthropogenic climate change has led to a rapid increase in awareness, interest and investment in carbon capture and storage (CCS). The recent global commitment to cease unabated coal power generation at COP26^1^ puts the spotlight on technologies such as CCS to provide the ability to use fossil fuels for energy production without the carbon emissions. Recent heavy public sector investment in CCS through the European Union Innovation Fund (€10 bn up to 2030^2^), the US ($12.5 bn as part of the Infrastructure Investment and Jobs Act^3^) and Norway ($1.8 bn for the Longship project^4^) demonstrates the global importance of CCS and therefore the value in work which supports its development.

The process of geological carbon storage (GCS) involves injection of, typically supercritical, CO_2_ into a subsurface reservoir rock in which it will remain for a geologically significant period of time as to positively offset the effect of anthropogenic emissions^5^. Geological formations must meet the broad following criteria to be deemed suitable for GCS: (i) host sufficient storage capacity in the pore structure; (ii) be conducive for fluid movement through the pore structure; (iii) offer effective characteristics (closure, sealing) for retaining injected CO_2_; and (iv) ideally possess suitable mineralogy to encourage mineral precipitation of carbon which traps it permanently. In this work we aim to assess four different sandstone units in terms of criteria (i) and (ii) through porosity and permeability analysis respectively.

Percolation theory is a method of describing how a network of nodes behave following the introduction of connections between these nodes, widely applicable in material science^6,7^. It can be applied to address a variety of challenges including scaling, through the definition of critical phenomena which are related to scaling laws^6–9^. Percolation theory was originally developed using mathematically random models applied to geometrically simple lattice structures^6^. The percolation threshold is defined as the minimum fraction of nodes required to exhibit connectivity in order for a pathway to form between two opposite boundaries of a structure^6^. This critical value has been the focus of many investigations owing to its importance for permeability in porous media^10–13^ as well as phase transition in thermodynamic systems and conductivity in magnetic and electrical systems^6^.

In this work we define an upper percolation threshold to describe the partially connected to fully connected regime transition in sandstones, described by Thomson et al.^14^. The upper percolation threshold, also known as the crossover porosity^15–17^, differs from the traditional percolation threshold in that it defines the point at which a network transitions from a state of partial connectivity to one of near full connectivity^14,18^. Partial connectivity is the regime in which the network is percolating but a significant proportion of available nodes are not connected to the percolating cluster. In contrast, the near fully connected regime is characterised by the bulk of available nodes being connected as one large percolating cluster. Meanwhile, the traditional percolation threshold is defined at a critical value where a partially connected network becomes completely disconnected and non-percolating^10–12,14,19^.

This work aims to give a definition of the upper percolation threshold using four different sandstone sample suites. Previous work on constraining the upper percolation threshold in sandstones has used a limited range of samples from a limited number of study sites^6,14,18^. Typically there is a focus on the traditional percolation threshold^6,10^. Whilst this is a valuable observation to make, definition of the upper threshold is also valuable, particularly in GCS reservoir characterisation where optimal connectivity is desirable. This definition of the upper percolation threshold in particular is of great benefit to a variety of fields and industries including hydrocarbon exploitation and aquifer management as well as carbon capture and storage. By defining a value at which near full network connectivity is achieved this effectively defines a key criterion for initial screening of a potential GCS reservoir. This value should be the minimum total porosity possessed by a given unit to qualify as a storage reservoir given that significant connectivity will allow for effective movement of injected fluid throughout the entirety of the reservoir. This brings benefits in using the full potential of a reservoir as well as favourable injectivity conditions.

In addition, we aim to use the porosity analysis in conjunction with permeability measurements to define a reliable porosity–permeability relationship in sandstones for this scale of measurement. An accurate relationship between these two key factors influencing reservoir suitability for GCS is very beneficial for initial evaluation of potential reservoirs, prior to full reservoir characterisation at larger scales. An effective relationship allows for measurements of porosity to be used to estimate a value of permeability, helping to assess the potentiality of the reservoir. Porosity measurements may be acquired through routine borehole logging as well as measurements made on core samples and cuttings. Such measurements are relatively straightforward and quick to make, unlike permeability measurements. Therefore, an accurate estimation of permeability from porosity alone is valuable when deciding whether further investigation is warranted of a particular site. Once zones of porosity are defined using core and geophysical log data, upscaled investigation can take place through a 3D seismic reflection survey allowing at least indicative volumetric analysis of rock mass available as storage space for carbon dioxide^20,21^.

Previous work on definition of the porosity–permeability relationship has shown that taking grain characteristics into account has a detrimental impact on the quality of the fit model when using a Kozeny–Carman based approach^22^. Consequently, in this work we use the suggested simpler approach, excluding grain characteristics, to constrain the relationship. Prior studies have attempted to use this same approach for the same purpose^14^ however, a relatively small number of samples from a limited number of study sites have been used. Therefore, we aim in this work to strengthen our understanding of the porosity–permeability relationship using a larger sample suite from multiple locations.

In addition to characterisation of materials in terms of porosity and connectivity it is useful to understand the controls on these two characteristics. Through the use of pore network models (PNMs), a popular method for characterising pore geometries^23–27^, this work aims to determine the relative influence of pores and throats on the connectivity and porosity of the study samples. Previous work using limited sample suites suggests that larger pores and throats contribute to greater porosity and in particular contribute to greater connected porosity^14,18,23^. We aim to validate these findings by using a larger number of samples from multiple locations and therefore assess the validity with regards to application beyond a small number of sampled localities.

In order to carry out this pore scale assessment we use a fully digital workflow based on digital image analysis (DIA) of pore scale images acquired using X-ray micro computed tomography (μCT)^18^. Traditional porosity and permeability analysis depends on laboratory techniques such as helium pycnometry and core flooding which require significant sample preparation, time and cost^14,28–32^. The digital workflow used here aims to demonstrate that reliable and meaningful results can be produced using this technique, which is increasing in popularity^14,24,28,33–35^, with the added benefit of being non-destructive, entirely digital in nature, easily repeatable and relatively inexpensive.

Previous work on the upper percolation threshold and porosity–permeability relationship using digital techniques^14^ uses relatively few samples, leaving room to improve the certainty in the resulting critical values and relationships. This study aims to improve the confidence in the definition of both the upper percolation threshold and porosity–permeability relationship by incorporating a large number of samples from different geographic locations and stratigraphic units. This will enable the proposal of an upper percolation threshold and porosity–permeability relationship which are applicable to sandstones from other study areas for the purpose of initial site screening, helping to carry out effective characterisation of potential GCS reservoirs.

## Methods

### Samples

**Figure 1:**
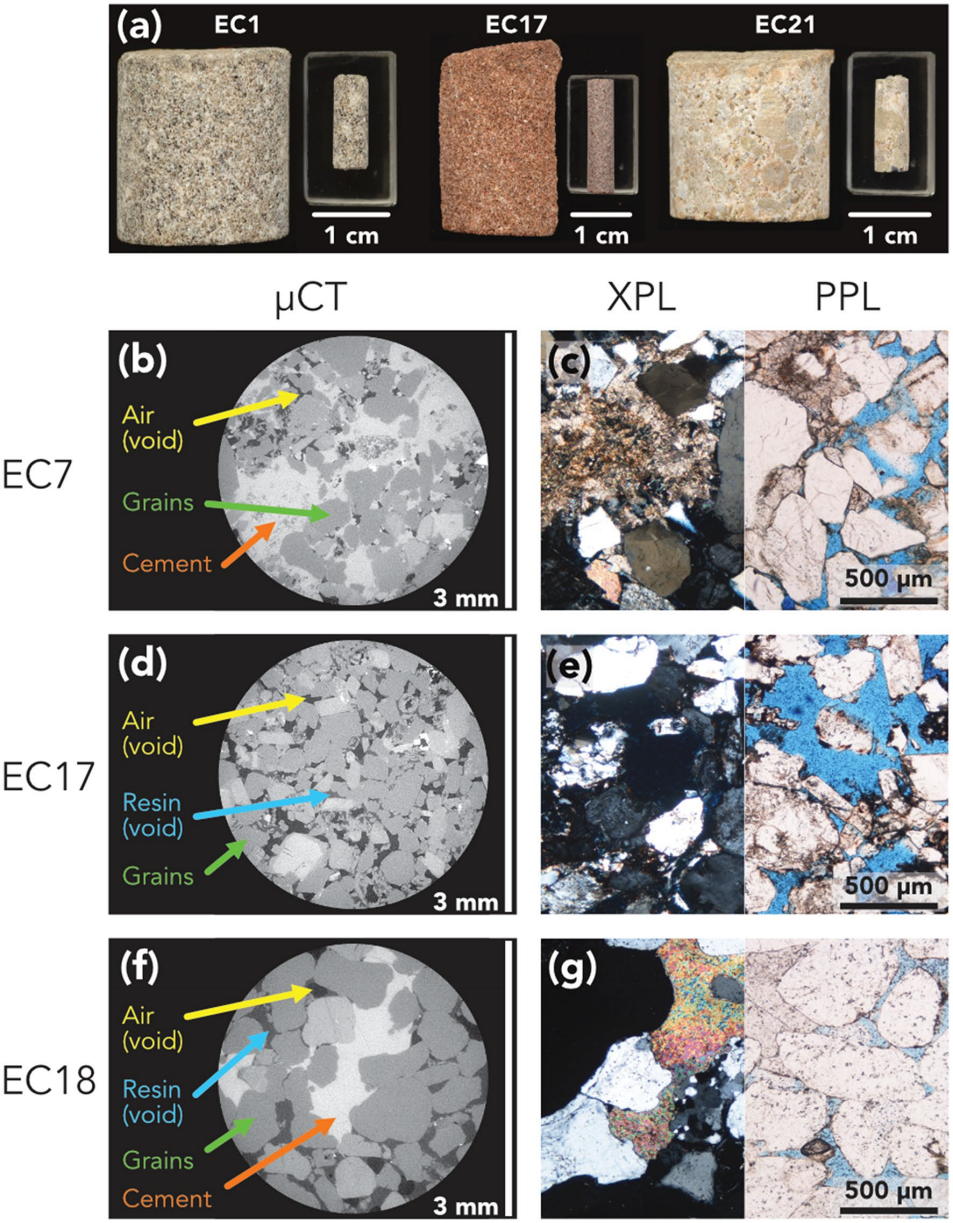
Examples of the material used for imaging and the resulting μCT and optical microscopy images in both XPL and PPL. (**a**) Shows the cut plug and mini-plug used for μCT imaging from three samples: EC1, EC17 and EC21, showing the range of materials from the English Channel sample suite. (**b**–**g**) show μCT images with phases labelled and XPL and PPL optical microscopy images of samples EC7, EC17 and EC18 respectively. In the PPL images the blue phase is a dyed resin to facilitate identification of the pore space.

Multiple sandstone samples have been collected from each of five offshore boreholes in the English Channel. A series of sandstone plug samples were acquired from the Otter Sandstone Formation (Sherwood Sandstone Group, English Channel) for the purpose of this study. These new samples are used alongside three other sample suites (i.e., the Wilmslow Sandstone Formation, Sellafield, UK^18^; Minard Formation, Porcupine Basin, N. Atlantic^22^ and Scottish Middle Coal Measures Formation, Glasgow, UK^18^) which have been previously studied by the associated authors. We used the same preparation and scanning process when working with the English Channel (EC) samples as has already been described for the other previously prepared materials^14,18,22^. Initial plugs acquired from the core material are typically ca. 2 cm in diameter and up to 10 cm long. These plugs were cut into mini plugs measuring 5 mm in diameter and 10 mm in length before undergoing X-ray micro computed tomographic (μCT) imaging. For a summary of the materials used in this work in terms of their geological context and imaging parameters we refer the reader to the Supplementary Information (Table S1). Figure 1 shows the EC sample μCT phases and supporting petrographic thin section photos of three samples.

### Image processing

We followed the methodology previously described for the Sellafield (SF), Glasgow (GG) and Porcupine Basin (PB) samples in order to process the acquired μCT images of the English Channel (EC)^14,18,22^. Using the commercial software package PerGeos (v1.7.0) a subvolume was extract from each 3D reconstruction to remove external voxels and any beam hardening artefacts which could not be satisfactorily removed using filtering. We used a non-local means filter to enhance the contrast between phases and remove background noise across the images prior to segmentation^36,37^.

### Porosity and permeability measurements

To allow our results to be directly comparable, we used the workflow described for the SF, GG and PB samples^14,18,22^ to make measurements on the EC samples. Due to the variety of phases present which appeared similar in some regions of the μCT images (Fig. 1) we used manual threshold segmentation. Segmentation was performed with the aid of dyed petrographic thin sections (Fig. 1) to accurately segment the pore space from other dark phases. Segmentation accuracy is a common point of contention in image analysis^38^ owing to the direct affect that it has on subsequent measurements^39^. Whilst automatic algorithms may be used to perform segmentation, typically a manual approach is favoured owing to the ability to fine tune the thresholds between phases^14,23,40–42^. This comes at the cost of direct comparability between samples and studies, resulting from operator bias and variation.

The resulting segmented volume fraction, designated as the pore space, was measured as a fraction of the total sample volume to determine total porosity. A connectivity algorithm allowed isolation of the pore space which facilitates connectivity throughout the entire sample between all external faces of the cuboidal subvolume. Measurement of this pore space as a fraction of the entire sample volume provided the connected porosity. For 3D volume renderings of the pore structures of the Otter Sandstone Formation samples, we refer the reader to the Supplementary Information. The measurements of porosity acquired using this technique are limited by the voxel size of the reconstructed 3D image. A degree of porosity will be unresolvable using μCT, such as nano porosity, and therefore, is not included in these measurements. Consequently, the measurements made must be considered as macro porosity at this scale.

We used a finite volume numerical model to solve the Stokes flow equations:

1 $$\nabla {\varvec{u}}=0,$$

2 $$- \nabla P+\mu {\nabla }^{2} {\varvec{u}}=0$$

where ${\varvec{u}}$ is velocity, $P$ is pressure and $\mu$ is fluid viscosity. We used ${10}^{-6}$ as the error tolerance for the convergence of the L_2_ norm of the residuals as has been previously recommended^41^. Application of Darcy’s Law to the velocity field solution allows determination of permeability. Permeability measured in this way must be considered as a maximum permeability owing to the inability of pore-lining phases such as illite and chrloite to be considered. The μCT image voxel size may not be sufficiently small to make identifying or distinguishing these phases from the bulk granular material possible.

### Pore geometry

Characterisation of the pore geometry was performed using pore network models (PNMs). PNMs are generated using a series of algorithms which approximate the pore structure as balls and sticks representing pores and throats respectively. In this work we applied the algorithms described by Youssef et al.^43^ hosted within the software package PerGeos, summarised here. A skeletonisation process generates a one-voxel-thick skeleton through the centre of the pore structure. Thresholds are defined where junctions form in the skeleton to identify dead ends, pore lines and throat lines. Pores and throats are classified based on the ratio of line length and maximum radius where a greater length to radius ratio results in classification as a throat^18^. The skeleton can then be grown again to fill the true pore structure, showing where one pore or throat ends and another begins. The PNMs may be interrogated to obtain measurements of both pores and throats. Coordination number is given as the number of other pores that a given pore is directly connected to via a throat. Pore radius is the maximum radius of a sphere which can fit within a given pore. Throat radius is measured in the same way as pore radius whilst throat length is the length of the skeletonisation line which passes through a given throat. As the Porcupine Basin material had not previously undergone PNM analysis, we report the results in this work alongside those from the English Channel samples.

## Results

### Porosity

**Figure 2:**
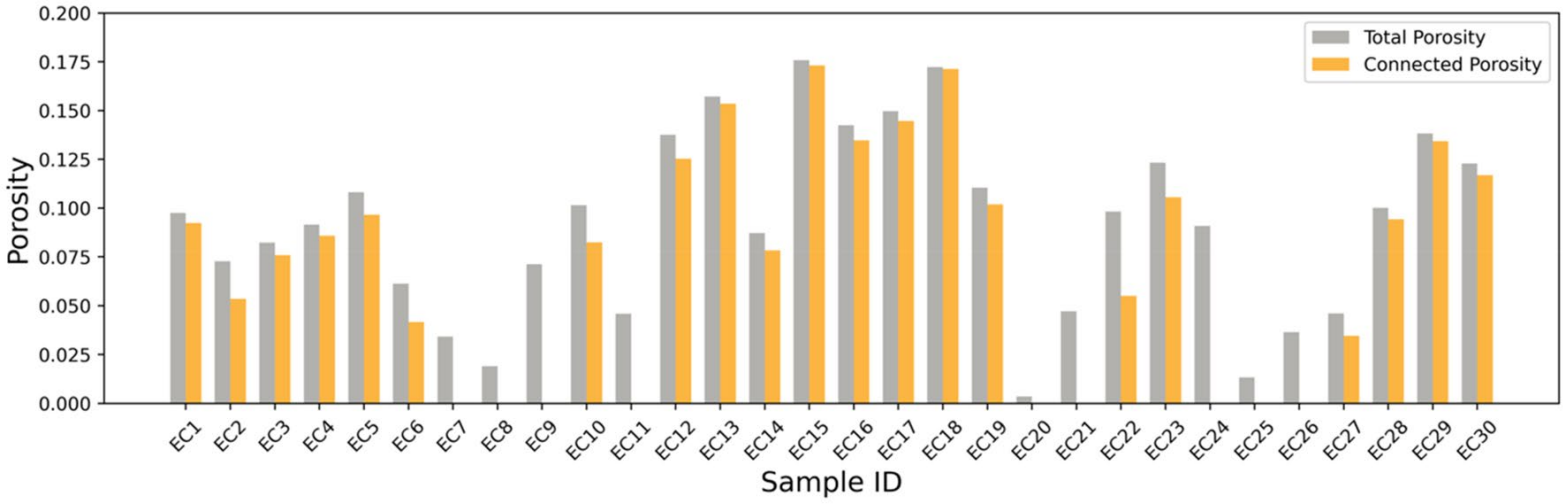
Bar chart showing the range of total and connected porosities across the Otter Sandstone Formation samples collected from the English Channel. A full range of degrees of connectivity are shown from near full connectivity through partial connectivity and no connectivity.

Total porosity measurements of 30 samples from the English Channel, pertaining to the Otter Sandstone Formation, exhibit a wide range between 3.5 and 17.6%, shown in Fig. 2 and Table 1. The mean value of total porosity is 9.1% across this sample suite, providing a set of measurements which fill the porosity range of 5–15% porosity which is left sparse by the samples previously studied by Payton et al.^18,22^, included in this culminative work.

**Table 1:** Porosity, connected porosity and permeability measurements of each study sample from the English Channel.

Sample ID	Depth (ft)	Total porosity (%)	Connected porosity (%)	Permeability (mD)
EC1	6113.8	9.8	9.2	97
EC2	6125	7.3	5.4	8
EC3	6135.9	8.2	7.6	41
EC4	6150.7	9.1	8.6	110
EC5	6152	10.8	9.7	28
EC6	6155.7	6.1	4.2	5
EC7	6278.8	3.4	0.0	0
EC8	6283.3	1.9	0.0	0
EC9	6293.8	7.1	0.0	0
EC10	6299.1	10.1	8.2	83
EC11	6310.2	4.6	0.0	0
EC12	6315	13.8	12.5	163
EC13	1638.5	15.7	15.3	288
EC14	1652.1	8.7	7.8	89
EC15	1669.6	17.6	17.3	512
EC16	1699.2	14.2	13.5	136
EC17	1728.2	15.0	14.5	387
EC18	1760.4	17.2	17.1	4655
EC19	6431.5	11.0	10.2	101
EC20	6451.9	0.4	0.0	0
EC21	6469	4.7	0.0	0
EC22	6476.8	9.8	5.5	69
EC23	6497.6	12.3	10.6	459
EC24	6530.4	9.1	0.0	0
EC25	6610	1.3	0.0	0
EC26	6625.2	3.6	0.0	0
EC27	6645.5	4.6	3.5	11
EC28	6663.9	10.0	9.4	49
EC29	6698.2	13.8	13.4	181
EC30	6731.4	12.3	11.7	103

Measurements from the other sample suites are available in their respective publications^18,22^.

Comparison of the total and connected porosities (Fig. 2) highlights that in some cases nearly the entirety of the porosity provides connectivity, such as in samples EC13, EC15 and EC18. Generally, as porosity reduces the proportion of the porosity which is connected diminishes, shown by the contrast between bars in samples EC2, EC6 and EC22. Additionally, there are a number of samples (e.g. EC7, 21 and 26) which display no connected porosity at all. These are typically those samples exhibiting much lower porosities.

**Figure 3:**
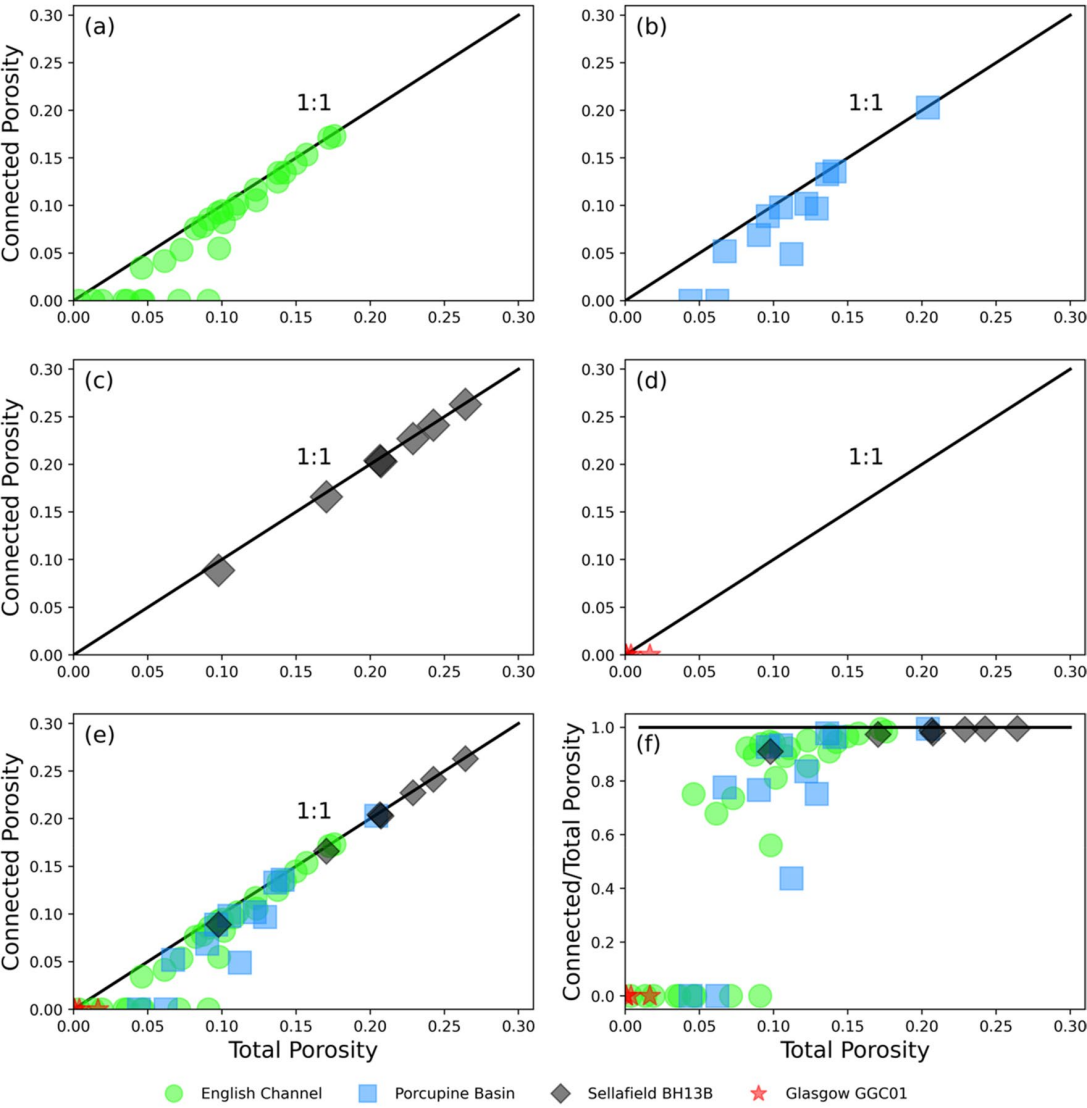
Plots showing the relationships between total and connected porosity across the range of study materials. (**a**) Shows results from the new English Channel study samples alone whilst (**b**) Show results measured by Payton et al.^22^ from the Porcupine Basin. (**c**, **d**) Show the results measured by Payton et al.^18^ from the Wilmslow Sandstone Fm. And Scottish Middle Coal Measures Fm. Respectively. The relationship across all study suites is shown in (**e**, **f**) which highlights the diversion of data points below the 1:1 relationship representing full connectivity. This diversion is the upper percolation threshold at ca. 14% total porosity. A region of partially connected data points are observed to then overlap with the lower, disconnected data points making the traditional percolation threshold difficult to define.

**Figure 4:**
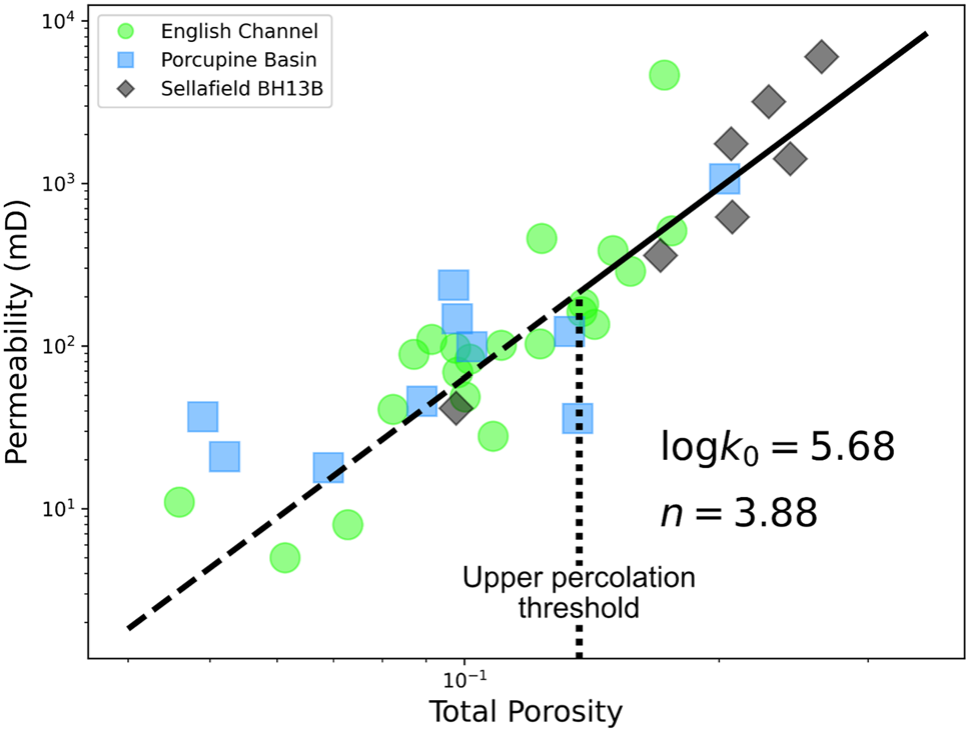
Porosity–permeability relationship plot on log axes, showing data from all sample suites. It is clear that the bulk of data points are found at > 10% total porosity, above the region of the upper percolation threshold. This results in a well characterised relationship in this region, leaving a more poorly defined range from 0 to 10% which requires further investigation. The vertical black broken line represents the 14% upper percolation threshold. The sloping black fit line is calculated using the fit parameters shown on the plot to provide a relationship given by, $K={10}^{5.68} {\phi }^{3.88}$. The solid part of the fit line represents the data range above the upper percolation threshold where this relationship is well constrained. The dashed part of the fit line represents the range below the upper percolation threshold where this fit becomes limited in its applicability.

The relationship between total and connected porosity across each sample suite can also be seen clearly in Fig. 3, where data from the English Channel (Fig. 4) is shown alongside that from the Porcupine Basin (Minard Formation; Fig. 3b), Sellafield BH13B (Wilmslow Sandstone Formation; Fig. 3c) and Glasgow GGC01 (Scottish Middle Coal Measures Formation; Fig. 3d). Figure 3e shows this relationship in all samples. It is apparent that at greater total porosities, above ca. 14%, that there is close to a 1:1 relationship between the two types of porosity, indicating that there is near full connectivity in the pore structure. In contrast, as total porosity reduces below ca. 14% data points can be observed to fall further below the 1:1 relationship line, indicating that a proportion of the porosity is becoming disconnected. Figure 3f shows this same phenomenon more clearly as data points fall below the horizontal line representing a 1:1 relationship. This transitional point at ca. 14% total porosity is the critical upper percolation threshold at which the pore structure moves from a near fully connected to a partially connected regime.

Several of our study samples exhibited no connected porosity despite varying amounts of total porosity (Figs. 2 and 3). This results in a cluster of data points at the bottom of Fig. 3d–f, isolated from the other data. These data points span a range of 0.3 to 9% total porosity. This range overlaps with a handful of data points which exhibit partial connectivity, showing conflict between where a regime of partial connectivity ends and a regime of completely isolated porosity begins.

### Permeability

Measurements of permeability across all sample suites exhibit a clear positive relationship with total porosity (Fig. 4). Permeability in the English Channel samples showing connected porosity, ranges from 5 to 4655 mD. These measurements span a significant range of porosity overlapping with both the Porcupine Basin and Sellafield materials, showing typically lesser and greater amounts of permeability respectively.

Critically, most data points shown in Fig. 4 exhibit porosities of > 10%, with only a minor number filling the > 0–10% porosity range. This results in an improved degree of confidence in the definition of the porosity–permeability relationship at greater porosities above the upper percolation threshold identified in Fig. 3. We calculated a linear fit (Fig. 4, black line) to the data, using fit parameters of $\mathrm{log}{10}_{{k}_{0}} = 5.68$ and $n=3.88$. Using the Kozeny–Carman equation we obtain a relationship between porosity and permeability in our sample suites according to, $K={10}^{5.68} {\phi }^{3.88}$, where $K$ is permeability and $\phi$ is total porosity. However, the proposed fit line is not suitable for porosities below the upper percolation threshold as below this point permeability will more rapidly decline with a steeper gradient. Therefore, the results presented here from a range of sandstones provide a good constraint on the porosity–permeability relationship for only the near fully connected materials which may be applied to other sandstone samples.

### Pore geometry characterisation

**Figure 5:**
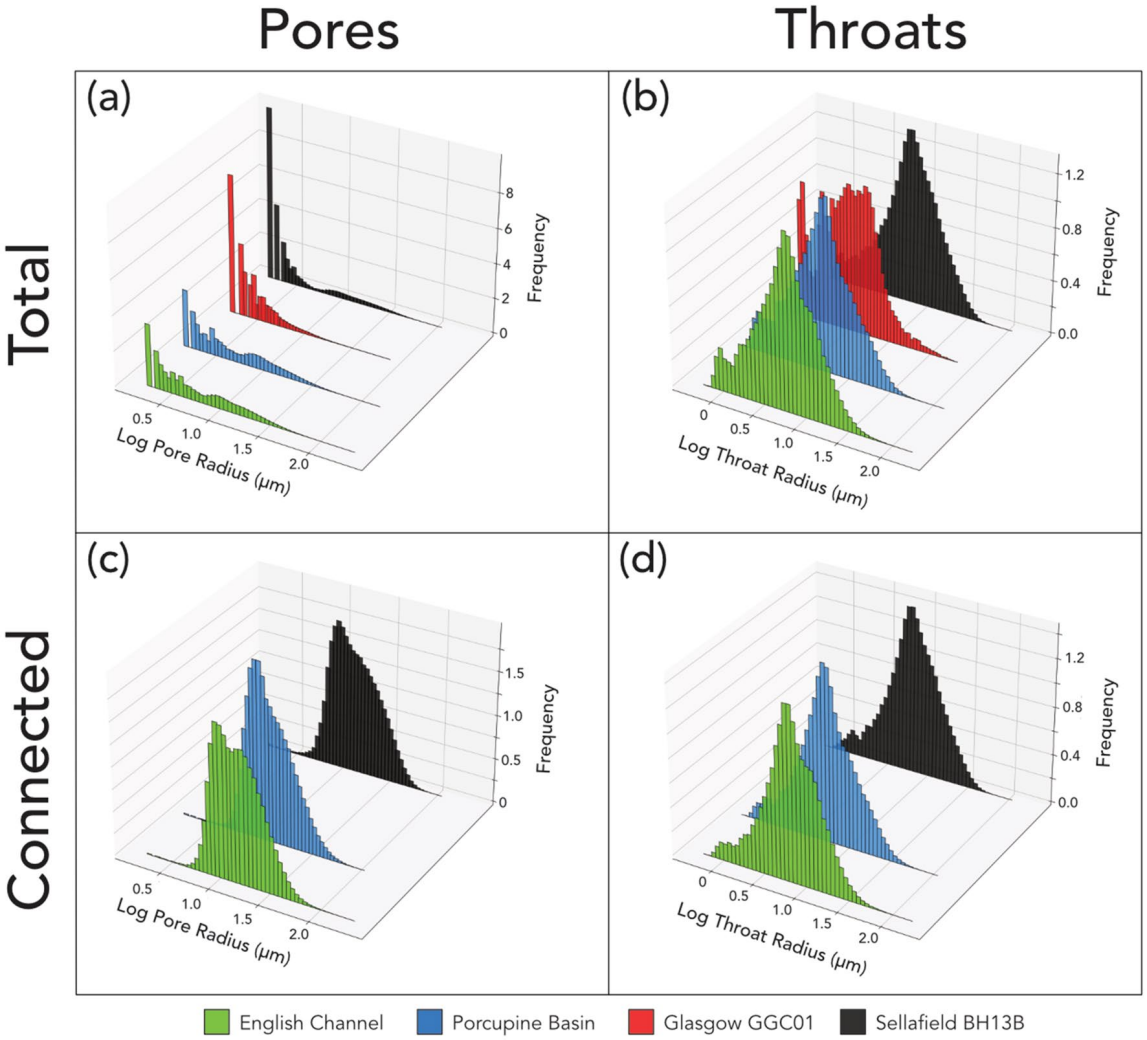
3D histograms showing the size distributions of pore and throat radius across all sample suites for both total and connected porosity. (**a**) Shows a large proportion of small pores with a secondary minor peak of larger pores, identified clearly in (**c**) where only connected porosity is considered. Observation of the connected pores alone shows that the smallest pores do not contribute to connectivity. (**b**) Shows a more normal distribution of the throat radii with a minor peak representing smaller throats. This minor peak is lost when observing only the connected throats (**d**) whilst the larger throats remain. Both the trends shown by pores and throats are similar in nature however, is far more dramatic in the case of the pores.

Interrogation of the pore network models (PNMs) produced for each study sample allows for the investigation and comparison of pore and throat characteristics (Table 2) and how they influence porosity. Figure 5 shows normalised histograms of pore and throat radii for both the total and connected porosity amongst all four sample suites. Figure 5 shows that a large number of pores with very small radii dominate the plotted distribution in all four cases. In addition to the dominant peak there are secondary minor peaks around log pore radii values of 1 in all sample suites other than Glasgow.

**Table 2:** Mean measurements of coordination number, pore radius, throat radius and throat length from pore network models of each English Channel and Porcupine Basin samples.

Sample ID	Coordination number	Pore radius (μm)	Throat radius (μm)	Throat length (μm)
EC1	3.7	19.9	10.1	63.8
EC2	4.5	15.6	8.1	49.2
EC3	3.7	18.7	9.4	59.3
EC4	3.6	20.3	10.7	63.7
EC5	4.1	18.1	9.1	53.5
EC6	3.8	19.4	10.9	62.2
EC7*	0.9	7.4	7.8	43.8
EC8*	0.8	7.2	9.2	48.6
EC9*	0.7	7.4	11.6	47.7
EC10	3.3	28.4	16.7	81.1
EC11*	0.9	7.0	8.3	38.7
EC12	3.5	20.7	10.5	61.7
EC13	4.2	17.9	8.7	56.7
EC14	4.2	22.0	12.3	73.0
EC15	4.2	18.6	8.8	60.6
EC16	3.8	18.7	9.7	60.4
EC17	3.9	20.7	10.9	65.9
EC18	4.6	24.3	14.1	90.5
EC19	3.7	18.5	9.2	61.1
EC20*	0.2	3.3	4.5	23.6
EC21*	0.7	6.5	9.1	52.8
EC22	3.6	21.9	11.4	63.5
EC23	3.8	23.9	13.5	74.2
EC24*	0.9	9.1	10.3	49.9
EC25*	0.4	4.3	5.2	27.2
EC26*	0.9	6.9	7.1	39.8
EC27	4.1	15.7	8.1	47.1
EC28	4.6	18.1	9.1	55.5
EC29	4.3	17.2	8.4	52.8
EC30	3.4	17.3	8.2	53.9
PB01	5.0	17.1	9.2	55.5
PB02	4.1	15.0	8.8	45.0
PB03	3.9	19.0	11.9	49.3
PB04*	1.2	5.1	8.2	32.7
PB05	4.7	24.7	16.5	56.7
PB06	3.3	14.2	7.0	45.0
PB07	4.7	13.9	7.4	40.0
PB08	3.9	13.5	6.4	43.6
PB09*	1.1	6.0	6.0	34.6
PB10	4.0	26.4	17.3	63.8
PB11	5.5	12.6	6.0	37.4
PB12	5.2	14.0	7.7	40.0

*Indicates measurements made on total porosity due to no connected porosity. Measurements from the other sample suites are available in their respective publications^18^.

Figure 5c also shows the pore radii size distribution but only for the pores contributing to sample connectivity. The results show that the bulk of smaller pores are not observed in this case, signifying that the smaller pores are not contributing to connectivity. The secondary minor peaks (Fig. 5a) remain, indicating the importance of the larger pores in providing connectivity. The lack of larger pores in the Glasgow samples appears to result in no connectivity.

In the same way, throat radii of each sample suite are examined in terms of total porosity in Fig. 5b. Compared to the size distribution of the pores the overall distribution appears to resemble a somewhat normal distribution. In each case a minor secondary peak can be observed around the region of smaller throat radii, especially in the Glasgow suite.

Figure 5d shows the throat radii size distribution for only the throats contributing to connectivity. In a similar fashion to the connected pores (Fig. 5c) the smallest throats are lost, leaving a smoother normal distribution. The loss of smaller throats is far less apparent than the loss of smaller pores. This is observed in all sample suites other than Glasgow which possesses no connectivity, predominantly due to the lack of larger pores.

**Figure 6:**
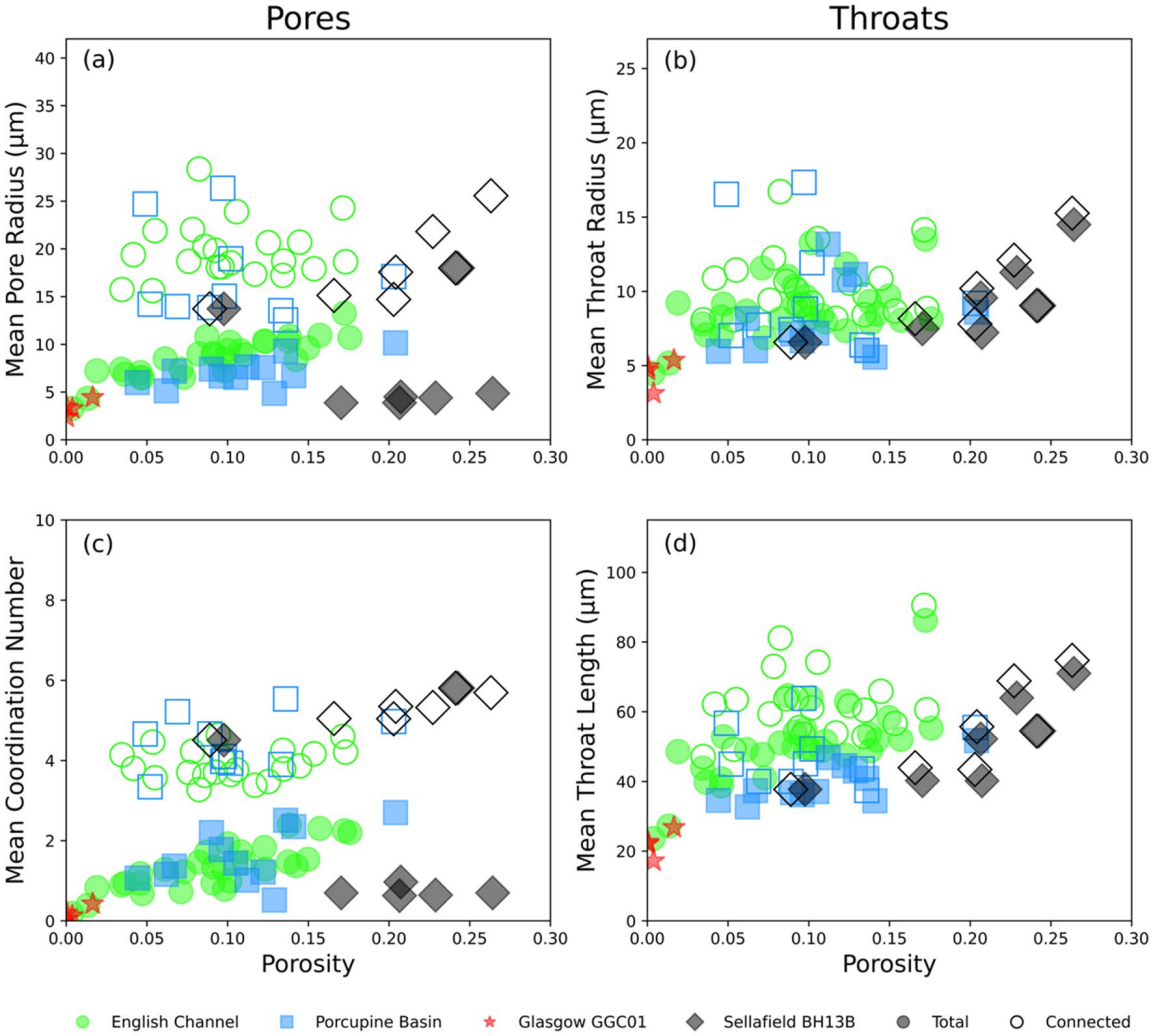
Plots comparing the mean pore and throat radii, coordination number and throat length across all sample suites against porosity. Hollow data points represent connected porosity measurements whilst filled data points represent total porosity measurements. Generally, each characteristic shows a positive relationship with porosity. When investigating the pore characteristics (**a**, **c**) there is a clear vertical displacement between the connected and total porosity measurements. This is not present when investigating the throat characteristics (**b**, **d**) where the two measurement types strongly overlap. The difference in vertical displacement between the pore and throat measurements indicates that the pores are apparently more influential on contributing to connectivity than the throats.

In addition to pore and throat radii we can measure the connectivity of each pore through the coordination number as well as the throat length using PNMs and evaluate each characteristic against porosity (Fig. 6). Figure 6a and c show the mean of these two pore characteristics for both total and connected porosity for each sample in all four suites, plotted against porosity. We can observe a global generally positive relationship between both mean pore radius and mean coordination number and porosity. The filled data points representing total porosity are notably positioned lower down the Y-axis as compared to the connected porosity (hollow) data points. This offset between the two porosities indicates that the greater mean pore radii and mean coordination numbers facilitate connectivity of the pore structures.

Figure 6b and d show the mean throat radius and length of each sample in each sample suite, evaluated against both total and connected porosity. Similarly, to the pores (i.e. Figure 6a,c), a global generally positive relationship is observed between porosity and both throat characteristics. Despite this similarity, the offset between the connected and total porosity data points is not observed so clearly when examining the throat measurements. This suggests that whilst a greater mean throat radius and length is preferable for promoting connectivity, it is less influential than the pore characteristics.

## Discussion

### The upper percolation threshold

The identification of a sandstone upper percolation threshold at ca. 14% total porosity is of key significance in the initial screening of siliciclastic materials and reservoirs. This value may be used as a criterion for determining reservoirs with suitable porous flow characteristics for geological carbon storage injection and hydrocarbon or aquifer extraction, prior to further full reservoir characterisation. Thomson et al.^14^ introduce the concept of three classifications of connectivity: near fully effective, partially effective and isolated. They define the upper percolation threshold between a near fully connected and partially connected regime at 10% total porosity. This study demonstrates through the use of a range of additional sandstones that this threshold manifests at a slightly higher total porosity value of 14% (Fig. 3). The inclusion of a range of samples from different study areas strengthens the reliability of this threshold for application to other sandstones.

Despite successful definition of the upper percolation threshold between a partially and near fully connected regime, the definition of the boundary between a fully isolated and partially connected regime remains unclear. As seen in Fig. 3, significant overlap between partially connected samples and fully isolated samples over a porosity range of 4.6–9% is present. Thomson et al.^14^ find no overlap in their samples and are able to define a threshold at 3% in agreement with other published estimations^10,13,44^. Using the study materials presented in this work we are unable to define a clear and certain threshold between these two regimes however, we can suggest a speculative definition.

All but three of our samples exhibiting porosity lacking any connectivity possess < 5% total porosity. Of the samples showing partial connectivity, the lowest total porosity is 4.6%. This allows us to speculate that the isolated–partial connectivity traditional percolation threshold is ca. 5% in sandstone. It is clear from this relative ambiguity than further work is required to focus on this region of transition from isolated to partially connected porosity. Definition of this threshold is challenging owing to the fact that available core material typically comes from high porosity intervals due to this being the main focus of the energy industry and is thus a biased sample set. However, as geological carbon storage (GCS) comes to the fore alongside ongoing work on containing subsurface radioactive waste and other contaminants, definition of the traditional percolation threshold is of equal importance when using the subsurface environment.

### The porosity–permeability relationship

In addition to accurate definition of a partially—near fully connected upper percolation threshold, the description of the porosity–permeability relationship is of significant benefit to the various fields of research and industry. We show that the relationship given by, $K={10}^{5.68} {\phi }^{3.88}$, provides a well constrained relationship between porosity and permeability, particularly where porosity is > 10% (Fig. 4). This clear relationship facilitates estimation of permeability with a reasonable degree of confidence from measurements of porosity alone. This provides the benefit of reducing the time and financial cost of testing permeability at the outset of a geological characterisation study. Implementation of the digital workflow used in this study and by others^14,18,41^ provides the additional benefit of being non-destructive and easily repeatable without the need for repeated sample preparation.

Despite reporting a porosity–permeability relationship which is applicable to other sandstones from other study sites, we acknowledge its weakness for materials with < 10% total porosity. The lack of data points here highlights the need for future work to focus on the > 0–10% porosity range with regards to permeability. The nature of the porosity–permeability positive relationship makes this a challenging problem to tackle. We suggest that additional imaging techniques may be necessary to best characterise materials in the > 0–10% porosity range. Whilst we were able to acquire μCT images with voxel sizes as small as 2.686 μm^3^, higher resolution images may enable identification of smaller scale porosity and connectivity which facilitates a degree of permeability. Techniques such as FIB-SEM, nano-XRM and fast synchrotron tomography have been used to investigate porosity typically in carbonates owing to the typical micro and nano scale pores present in these materials^32,45–47^. The Scottish Middle Coal Measures sandstone used in this study and investigated by Payton et al.^18^, may well benefit from using such techniques to interrogate the abundance of cement shown to severely inhibit porosity and permeability^18^. Whilst such imaging techniques have been used on sandstone for other purposes^48–51^, it would be beneficial to use these images to investigate the > 0–10% porosity relationship with permeability. This would allow for a better understanding of the full porosity–permeability spectrum, useful in other fields such as subsurface waste containment or CCS cap rock integrity.

### Pore and throat geometry

Through the comparison of pore and throat characteristics for both total and connected porosity (Figs. 5 and 6), we clearly show that the pore characteristics measured exert more of a control on the porosity than the throat characteristics. Across all four sample suites the mean pore radius of the total porosity is 7.7 μm whilst the mean pore radius of the connected porosity is 18.8 μm. This significant difference, shown graphically by the emphasis of the minor secondary peak in Fig. 5c as opposed to a, clearly highlights that larger pores are a key control on facilitating connectivity within a pore structure.

Meanwhile, Fig. 5b and d show that whilst there is a loss of narrower pore throats when comparing total to connected porosity it is not nearly as significant as in the pores. Across all four sample suites the mean throat radius increases from 8.4 (total) to 10.1 μm (connected). This far less significant change indicates that whilst larger throats are beneficial to facilitating connectivity, they are less influential than the pores.

These conclusions are supported when considering additional pore and throat characteristics in the form of coordination number and throat length respectively (Fig. 6). The significant offset between the connected and total porosity data points clearly present in the pore characteristics but absent from the throat characteristics reinforces that pore geometry is more crucial for facilitating connectivity than throat geometry.

### Implications for geological carbon storage

In addition to presenting relationships pertaining to porosity and permeability in these study samples which can be applied to other sandstones, we have provided a thorough porosity and permeability assessment. Therefore, we can provide a broad indication of each study suite’s suitability for further study in the context of geological carbon storage. As already described by Payton et al.^18^, the Scottish Middle Coal Measures appears to be entirely unsuitable for GCS owing to the extremely low porosity and lack of connectivity and therefore permeability. Whilst further investigation at smaller scales, as suggested in this work, may facilitate better understanding of the porosity–permeability relationship and traditional percolation threshold, this unit is completely outside the range of porosities suitable for GCS.

The Wilmslow Sandstone Formation presents the greatest degree of porosity and permeability amongst the studied sample suites. Just one sample exhibits clear partial connectivity whilst the remainder show near full connectivity. Consequently, further investigation into this unit as a reservoir for GCS would be worthwhile.

The Minard Formation sandstone pertaining to the Porcupine Basin exhibits an intermediate range of porosity with most samples showing total porosities below the defined 14% upper percolation threshold for near full connectivity. Consequently, the permeabilities of this material are also relatively intermediate, making it a reasonable option for further investigation however, in comparison to the Wilmslow Sandstone Formation it is less appealing and there are likely many more suitable options.

Finally, the Otter Sandstone Formation exhibits the widest range of both porosity and permeability. Many sample volumes recorded intermediate porosity values around the 14% upper percolation threshold, with a significant portion also showing no connectivity. This suggests that whilst some areas of this unit show great potential, in terms of porosity and permeability, as a storage reservoir there may be too much heterogeneity present to facilitate effective injection and storage. Despite this, we suggest that the area should be investigated further to identify if more homogenous intervals are present which exhibit the favourable characteristics observed in a portion of our sampled material.

## Conclusions

Using four varied sandstone sample suites, we have been able to propose an upper percolation threshold for the transition from partial to near full pore connectivity of 14% total porosity. This value is supported by the measurements of each sample suite. Due to a significant portion of data points overlapping which exhibit no connectivity and partial connectivity we are unable to propose a certain value of the traditional percolation threshold between fully isolated pores and partial connectivity. Based on the available results, we speculate that this threshold may be present at ca. 5% total porosity.

We report a porosity–permeability relationship according to, $K={10}^{5.68} {\phi }^{3.88}$, which we show to be effective in the total porosity region > 10%. Due to a lack of measurements in the > 0–10% porosity range we suggest that higher resolution imaging techniques may be required to constrain the relationship below the 14% upper percolation threshold.

Using pore network models, we have been able to show that the properties of pores, rather than throats, are the dominant controlling factor of connectivity within pore structures. Greater pore coordination number and pore radius facilitates greater connectivity far more significantly than greater throat radius or length.

Based on our porosity and permeability analysis we suggest that the Scottish Middle Coal Measures Formation is unsuitable for further investigation for geological carbon storage. Both the Otter Sandstone Formation and Minard Formation sandstone exhibit a range of porosities and permeabilities either side of the upper percolation threshold. The Otter Sandstone Formation shows the potential to host greater porosity, leading us to suggest that further investigation of this area may be beneficial in more homogenous sandy intervals. However, there are likely better options to investigate than the Minard Formation which exhibits only intermediate porosity and permeability. Finally, the Wilmslow Sandstone Formation displays the most favourable characteristics for a geological storage reservoir therefore, further investigation is strongly recommended.

## Supplementary Information

Supplementary Information.

## Data Availability

The μCT images used in this article are available from a variety of sources. Images of the Wilmslow Sandstone Fm. for samples with a SF prefix are available from Payton et al.^18^. Images of the Minard Formation Sandstone from the Porcupine Basin for samples with a PB prefix are available from Payton et al.^22^. Images of the Otter Sandstone Formation (Sherwood Sandstone Group) for samples with an EC prefix are available from the Royal Holloway, University of London Figshare repository, 10.17637/rh.19397912.

## Code availability

The code used to perform analysis and plot the output of the analyses is available alongside the measurement outputs in the Royal Holloway, University of London Figshare repository, 10.17637/rh.19398293.
