# Supplementary material for: The upper percolation threshold and porosity–permeability relationship in sandstone reservoirs using digital image analysis
Source: Sci Rep. 2022 Jul 4;12:11311. doi: 10.1038/s41598-022-15651-3 (PMC9253316; doi:10.1038/s41598-022-15651-3)
Supplement: Supplementary file 1 — Supplementary Information. [file 41598_2022_15651_MOESM1_ESM.docx]

**Supplementary Information**

for the article

**The Upper Percolation Threshold and Porosity-Permeability Relationship in Sandstone Reservoirs Using Digital Image Analysis**

published in

**Scientific Reports**

Ryan L. Payton^1,^*, Domenico Chiarella^1^ and Andrew Kingdon^2^

^1^ Royal Holloway, University of London, Department of Earth Sciences, Egham, Surrey, United Kingdom

^2^ British Geological Survey, Keyworth, Nottingham, United Kingdom

* Corresponding author: [ryan.payton.2015@live.rhul.ac.uk](mailto:ryan.payton.2015@live.rhul.ac.uk)

**Table S1.** Summary of all samples used in this work in terms of geology and imaging parameters. *Payton et al.^[22]^, **Payton et al.^[18]^.

| **Sample ID** | **Borehole ID** | **Location** | **Geological Formation** | **Image Voxel Size (μm^3^)** | **Study Subvolume (μm)** |
| --- | --- | --- | --- | --- | --- |
| EC1 | 98/11-2 | English Channel | Otter Sandstone Formation | 3.0 | 2007 x 2007 x 2487 |
| EC2 |  |  |  |  |  |
| EC3 |  |  |  |  |  |
| EC4 |  |  |  |  |  |
| EC5 |  |  |  |  |  |
| EC6 |  |  |  |  |  |
| EC7 | 98/11-3 |  |  |  |  |
| EC8 |  |  |  |  |  |
| EC9 |  |  |  |  |  |
| EC10 |  |  |  |  |  |
| EC11 |  |  |  |  |  |
| EC12 |  |  |  |  |  |
| EC13 | 98/06-9 |  |  |  |  |
| EC14 |  |  |  |  |  |
| EC15 |  |  |  |  |  |
| EC16 |  |  |  |  |  |
| EC17 |  |  |  |  |  |
| EC18 |  |  |  |  |  |
| EC19 | 98/11-1 |  |  |  |  |
| EC20 |  |  |  |  |  |
| EC21 |  |  |  |  |  |
| EC22 |  |  |  |  |  |
| EC23 |  |  |  |  |  |
| EC24 |  |  |  |  |  |
| EC25 | 98/11-4Z |  |  |  |  |
| EC26 |  |  |  |  |  |
| EC27 |  |  |  |  |  |
| EC28 |  |  |  |  |  |
| EC29 |  |  |  |  |  |
| EC30 |  |  |  |  |  |
| PB01* | 26/28-1 | Porcupine Basin, N. Atlantic | Minard Formation | 2.519 | 1687.73 × 1687.73 × 2350.23 |
| PB02* |  |  |  | 2.519 | 1697.81 × 1697.81 × 2350.23 |
| PB03* |  |  |  | 2.518 | 1684.54 × 1684.54 × 2354.33 |
| PB04* |  |  |  | 2.518 | 1709.73 × 1709.73 × 2354.33 |
| PB05* |  |  |  | 2.518 | 1709.72 × 1709.72 × 2354.33 |
| PB06* | 26/28-2 |  |  | 2.518 | 1709.73 × 1709.72 × 2354.33 |
| PB07* |  |  |  | 2.519 | 1709.72 × 1709.72 × 2354.33 |
| PB08* |  |  |  | 2.519 | 1709.72 × 1709.72 × 2354.33 |
| PB09* |  |  |  | 2.519 | 1709.72 × 1709.72 × 2354.33 |
| PB10* |  |  |  | 2.519 | 1709.72 × 1709.72 × 2354.33 |
| PB11* |  |  |  | 2.519 | 1687.73 × 1687.73 × 2350.23 |
| PB12* |  |  |  | 2.519 | 1697.81 × 1697.81 × 2350.23 |
| SF696** | SFBH13B | Sellafield, UK | Wilmslow Sandstone Formation | 2.6860 | 1590.12 × 2073.59 × 2575.88 |
| SF697** |  |  |  | 2.6861 | 1786.26 × 1909.82 × 2575.97 |
| SF698** |  |  |  | 2.6861 | 1737.91 × 1960.85 × 2575.97 |
| SF699** |  |  |  | 2.6861 | 1866.84 × 1834.61 × 2575.97 |
| SF700** |  |  |  | 2.6862 | 1829.3 × 1861.54 × 2576.07 |
| SF701** |  |  |  | 2.6862 | 1842.73 × 1850.79 × 2576.07 |
| SF702** |  |  |  | 2.8409 | 1971.58 × 1926.13 × 2724.42 |
| GG496** | GGC01 | Glasgow, UK | Scottish Middle Coal Measures Formation | 2.8409 | ﻿1497.15 × 1528.40 × 2724.42 |
| GG497** |  |  |  | 2.8409 | ﻿1843.74 × 2025.56 × 2582.38 |
| GG498** |  |  |  | 2.8410 | ﻿1929.04 × 2872.25 × 2826.80 |
| GG499** |  |  |  | 2.8410 | ﻿1971.65 × 1934.72 × 2724.52 |

**Figure S1.** 3D volume renderings of the total pore space within samples EC1-6 from well 98/11-2. The grey material represents the pore structure and empty space is where solid material is present. * The scale bar is applicable to all samples.


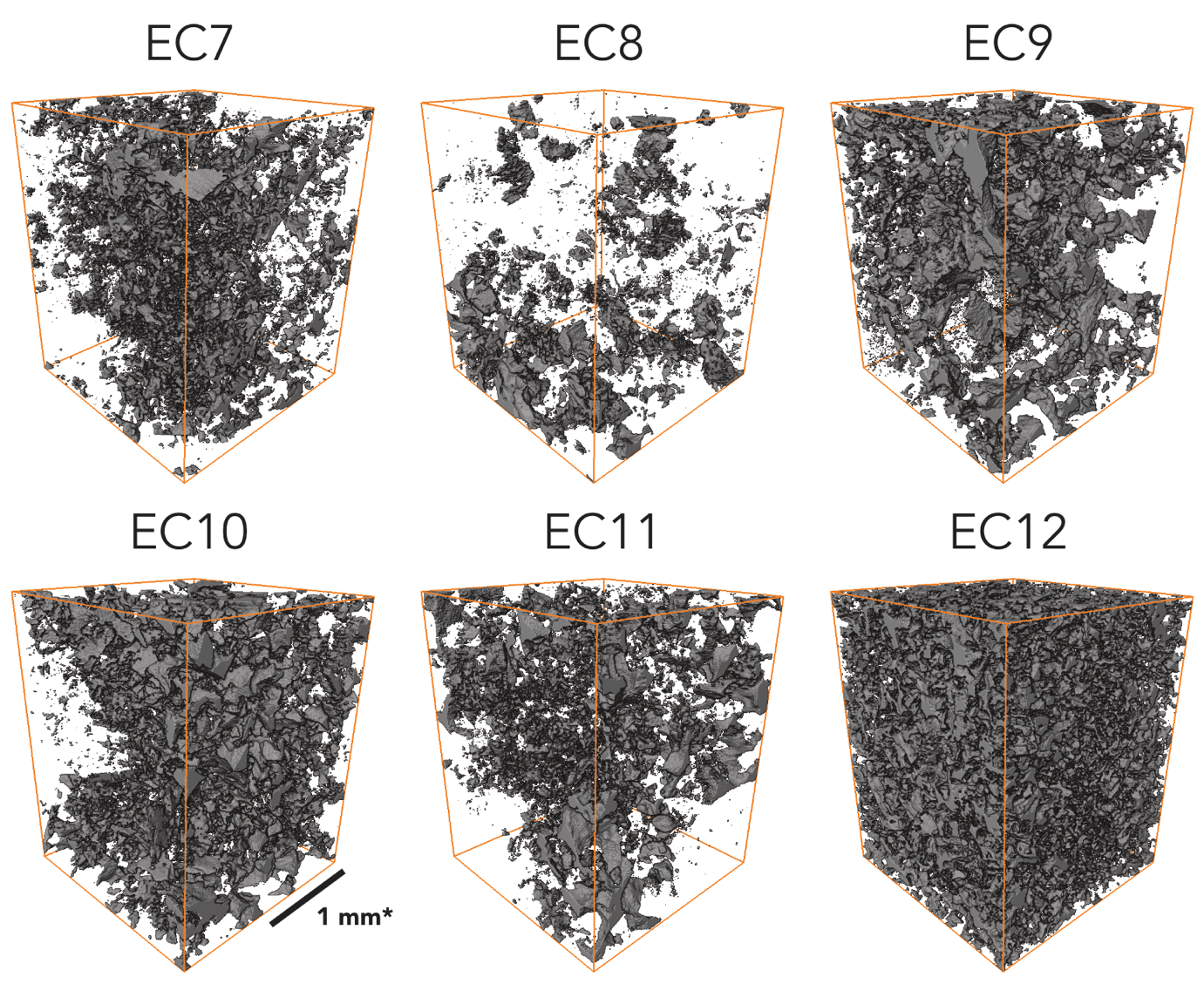


**Figure S2.** 3D volume renderings of the total pore space within samples EC7-12 from well 98/11-3. The grey material represents the pore structure and empty space is where solid material is present. * The scale bar is applicable to all samples.

**
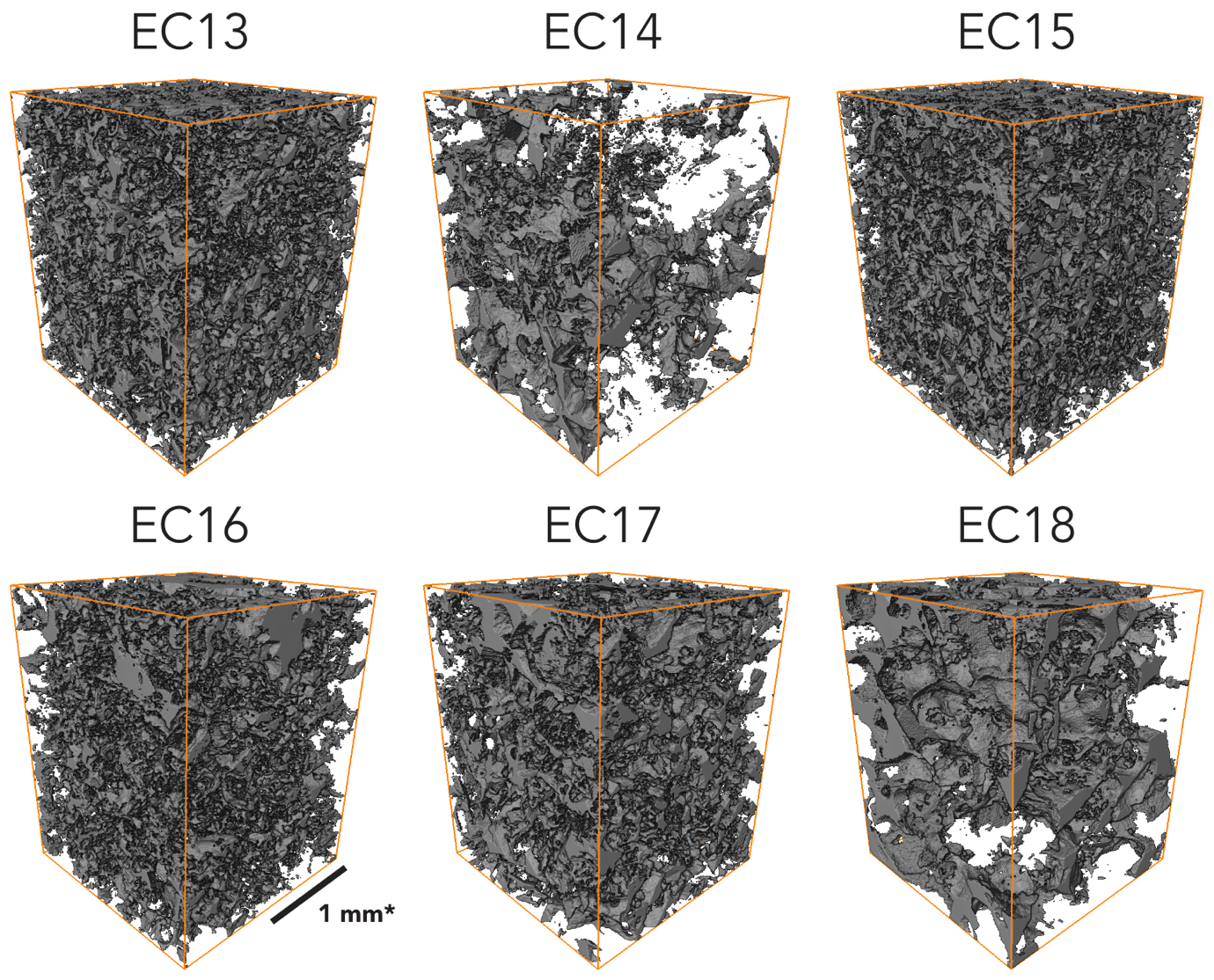
**

**Figure S3.** 3D volume renderings of the total pore space within samples EC13-18 from well 98/06-9. The grey material represents the pore structure and empty space is where solid material is present. * The scale bar is applicable to all samples.

**
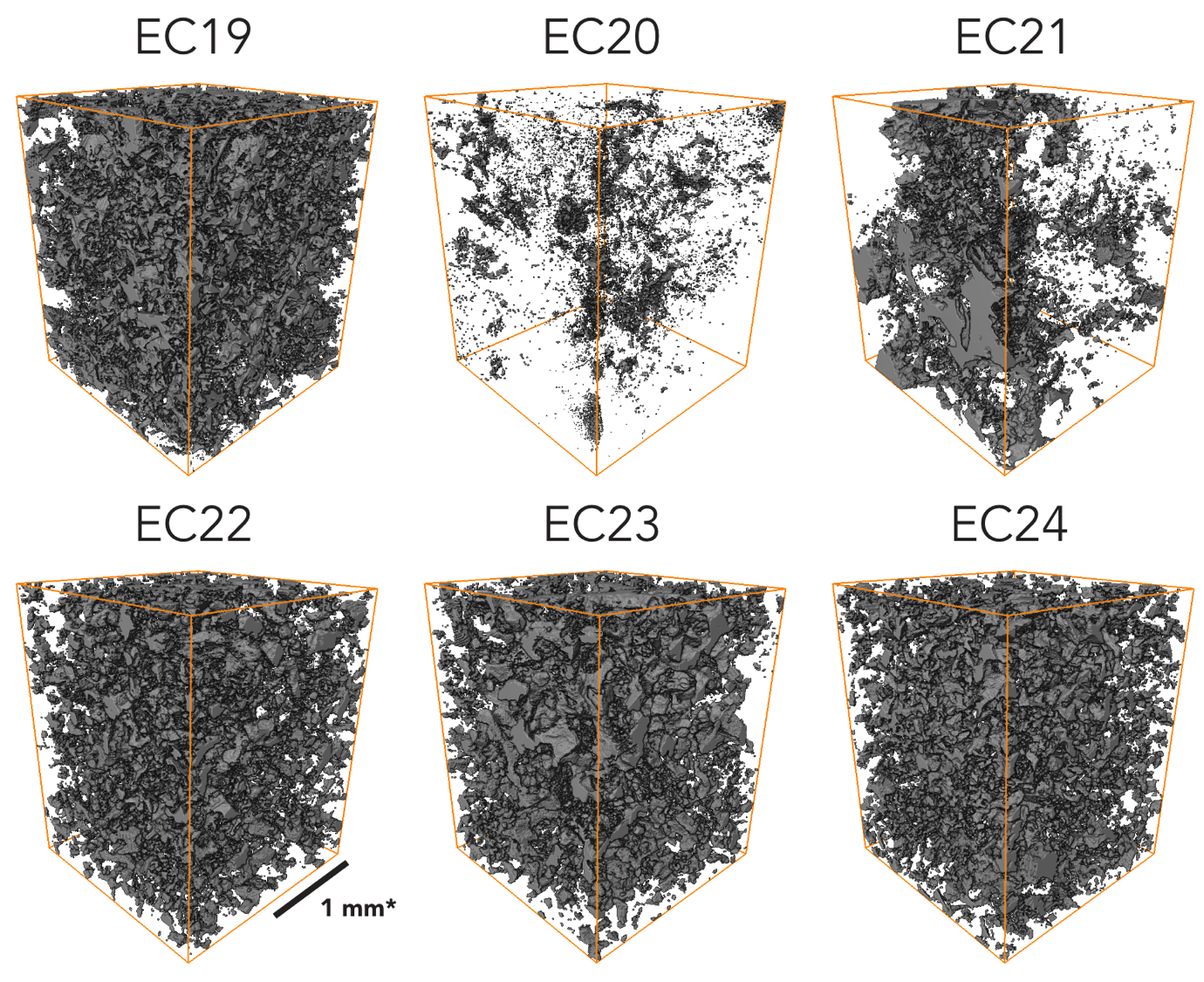
**

**Figure S4.** 3D volume renderings of the total pore space within samples EC19-24 from well 98/11-1. The grey material represents the pore structure and empty space is where solid material is present. * The scale bar is applicable to all samples.

**
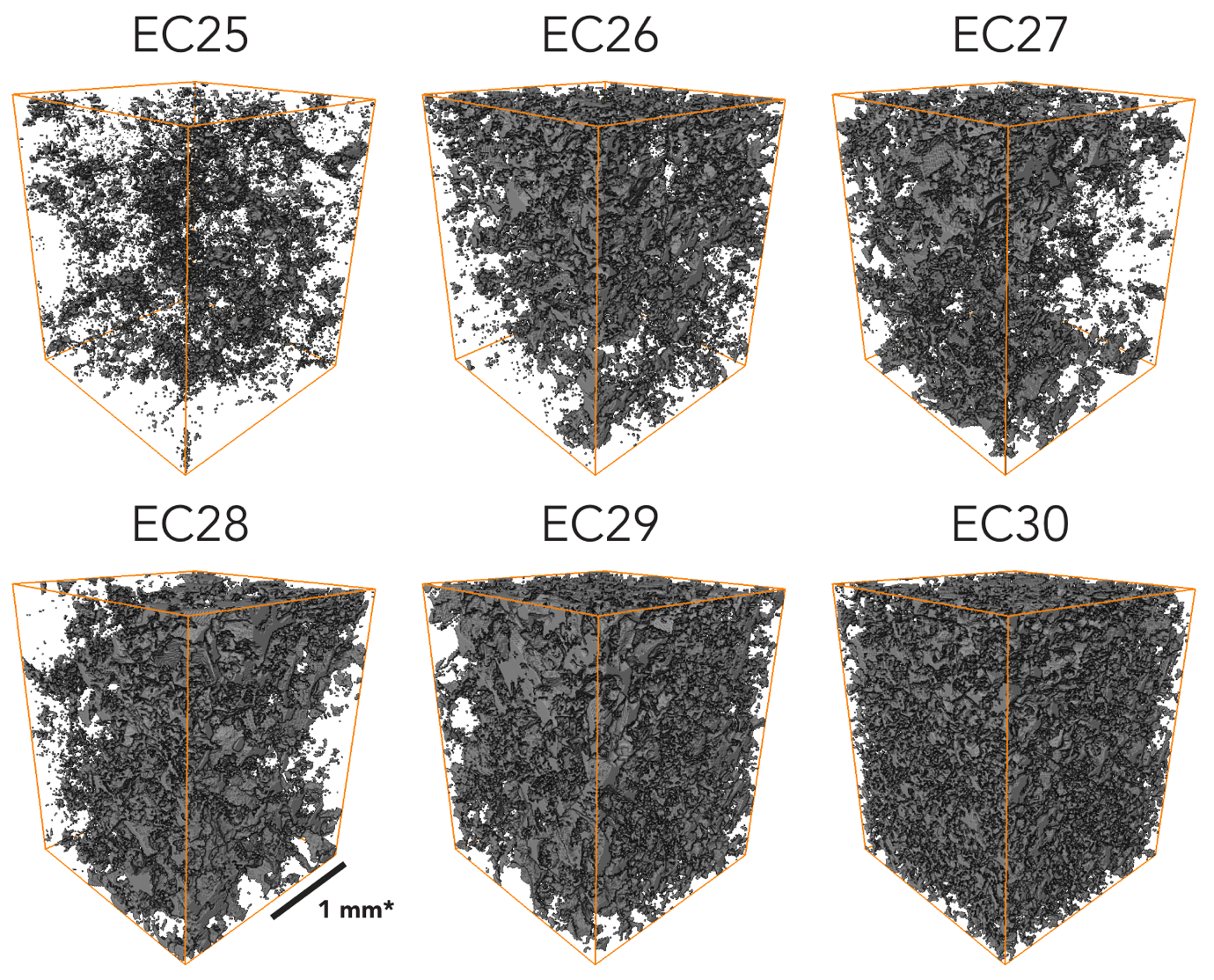
**

**Figure S5.** 3D volume renderings of the total pore space within samples EC25-30 from well 98/11-4Z. The grey material represents the pore structure and empty space is where solid material is present. * The scale bar is applicable to all samples.

**REFERENCE LIST – in accordance with the main body of the article**

18. Payton, R. L. *et al.* Pore-scale assessment of subsurface carbon storage potential: implications for the UK Geoenergy Observatories project. *Pet. Geosci.* **27**, (2021).

22. Payton, R. L., Chiarella, D. & Kingdon, A. The influence of grain shape and size on the relationship between porosity and permeability in sandstone: a digital approach. *Sci. Rep.* **12**, 7531 (2022).
